# SNX27 links DGKζ to the control of transcriptional and metabolic programs in T lymphocytes

**DOI:** 10.1038/s41598-017-16370-w

**Published:** 2017-11-27

**Authors:** M. Tello-Lafoz, C. Rodríguez-Rodríguez, G. Kinna, L. S. Loo, W. Hong, B. M. Collins, R. D. Teasdale, I. Mérida

**Affiliations:** 1 Department of Immunology and Oncology, Centro Nacional de Biotecnología (CNB-CSIC), Darwin 3, Campus UAM Cantoblanco, 28079 Madrid, Spain; 2 Institute for Molecular Bioscience, The University of Queensland, St. Lucia, Queensland, 4072 Australia; 3 School of Biomedical Sciences, The University of Queensland, St. Lucia, Queensland, 4072 Australia; 4 Institute of Molecular and Cell Biology A* STAR, Singapore, 138673 Singapore

## Abstract

Sorting nexin 27 (SNX27) recycles PSD-95, Dlg1, ZO-1 (PDZ) domain-interacting membrane proteins and is essential to sustain adequate brain functions. Here we define a fundamental SNX27 function in T lymphocytes controlling antigen-induced transcriptional activation and metabolic reprogramming. SNX27 limits the activation of diacylglycerol (DAG)-based signals through its high affinity PDZ-interacting cargo DAG kinase ζ (DGKζ). SNX27 silencing in human T cells enhanced T cell receptor (TCR)-stimulated activator protein 1 (AP-1)- and nuclear factor κB (NF-κB)-mediated transcription. Transcription did not increase upon DGKζ silencing, suggesting that DGKζ function is dependent on SNX27. The enhanced transcriptional activation in SNX27-silenced cells contrasted with defective activation of the mammalian target of rapamycin (mTOR) pathway. The analysis of *Snx27* ^−/−^ mice supported a role for SNX27 in the control of T cell growth. This study broadens our understanding of SNX27 as an integrator of lipid-based signals with the control of transcription and metabolic pathways.

## Introduction

The sorting nexins (SNX) are proteins that regulate intracellular protein trafficking and endosomal signaling. SNX27 is unique in the SNX family in that it has an N-terminal PSD-95, Dlg1, ZO-1 (PDZ) domain that binds proteins bearing a C-terminal class 1 PDZ-binding motif (PDZ-bm), a [S/T]-X-ϕ (ϕ = any hydrophobic residue) consensus motif^1,2^. The best-characterized SNX27 function is that of mediate the fast recycling of PDZ-bm-containing transmembrane receptors^3,4^, transporters^5^ and ion channels^6^. Recent studies have identified SNX27 high- and low-affinity ligands based on the amino acid sequences upstream of the PDZ-bm; high-affinity cargoes require acidic residues located at -3 and -5 positions that are able to clamp a conserved arginine on the SNX27 surface^7^.

In addition to transmembrane cargoes, SNX27 also interacts with the lipid kinase diacylglycerol (DAG) Kinase ζ (DGKζ)^8,9^. Biophysical and biochemical analyses of DGKζ C-terminal E-D-Q-E-T-A-V sequence demonstrated that it is a high-affinity ligand of SNX27^7^. DGKζ converts DAG lipids to phosphatidic acid (PA), a function that in T lymphocytes provides negative regulation of effector signals^10^. The recognition by the T cell receptor (TCR) of antigens presented by professional antigen presenting cells triggers phospholipase C-dependent DAG generation at the site of cell-cell contact termed immune synapse (IS). Local DAG production is followed by polarization and continuous recycling of DAG-enriched organelles^11^. Accumulation of DAG at the IS membrane stabilizes and activates several DAG-binding proteins including Ras guanyl nucleotide-releasing protein 1 (RasGRP1)^12^ and PKCα^13^, which link DAG generation to Ras/ERK activation, driving activator protein 1 (AP-1)-dependent transcription^14^. DAG also activates PKCθ that is selectively recruited to the IS by the costimulatory receptor CD28^15^. PKCθ recruiting and activation leads to nuclear factor κB (NF-κB)-dependent transcription^16,17^ and cooperates in the activation of the mammalian target of rapamycin complex 1 (mTORC1)^18^. DGKζ-mediated DAG consumption facilitates attenuation of these pathways downstream of TCR and CD28 triggering^19^.

SNX27 and DGKζ co-localize at the recycling compartment in resting T lymphocytes. Following antigen recognition SNX27-positive endosomes polarize to the immune synapse^8,9^, as has also been shown for DGKζ^20^. SNX27 silencing results in enhanced ERK phosphorylation, suggesting common functions with those of DGKζ^8^. Here we explored in more detail the consequences of SNX27 knockdown in DGKζ regulated T cell responses both in T cell cultures and genetically modified *Snx27* ^−/−^ mice. Our results demonstrate that SNX27-silencing in Jurkat T cells results in Ras/ERK/AP-1 and NF-κB pathway hyperactivation, the two main DAG-regulated pathways in T lymphocytes. Such activation did not increase further upon DGKζ silencing suggesting that constitutive interaction of SNX27 with DGKζ sustains its function as a negative regulator of DAG metabolism. Analysis of SNX27-deficient mice demonstrated normal T cell differentiation and enhanced CD69 activation in agreement with SNX27 and DGKζ acting on the Ras/ERK/AP-1 pathway. In contrast, SNX27 silencing in Jurkat cells decreased mTORC1-dependent S6 kinase (S6K) activation that correlated with limited antigen-induced cell growth of T cells from *Snx27* ^−/−^ mice. Our results extend the knowledge on SNX27 functions and suggest a previous unrecognized contribution on the coordinated regulation of DAG-based signals.

## Results

### Analysis of DAG-regulated signaling in SNX27-silenced activated T cells

**Figure 1:**
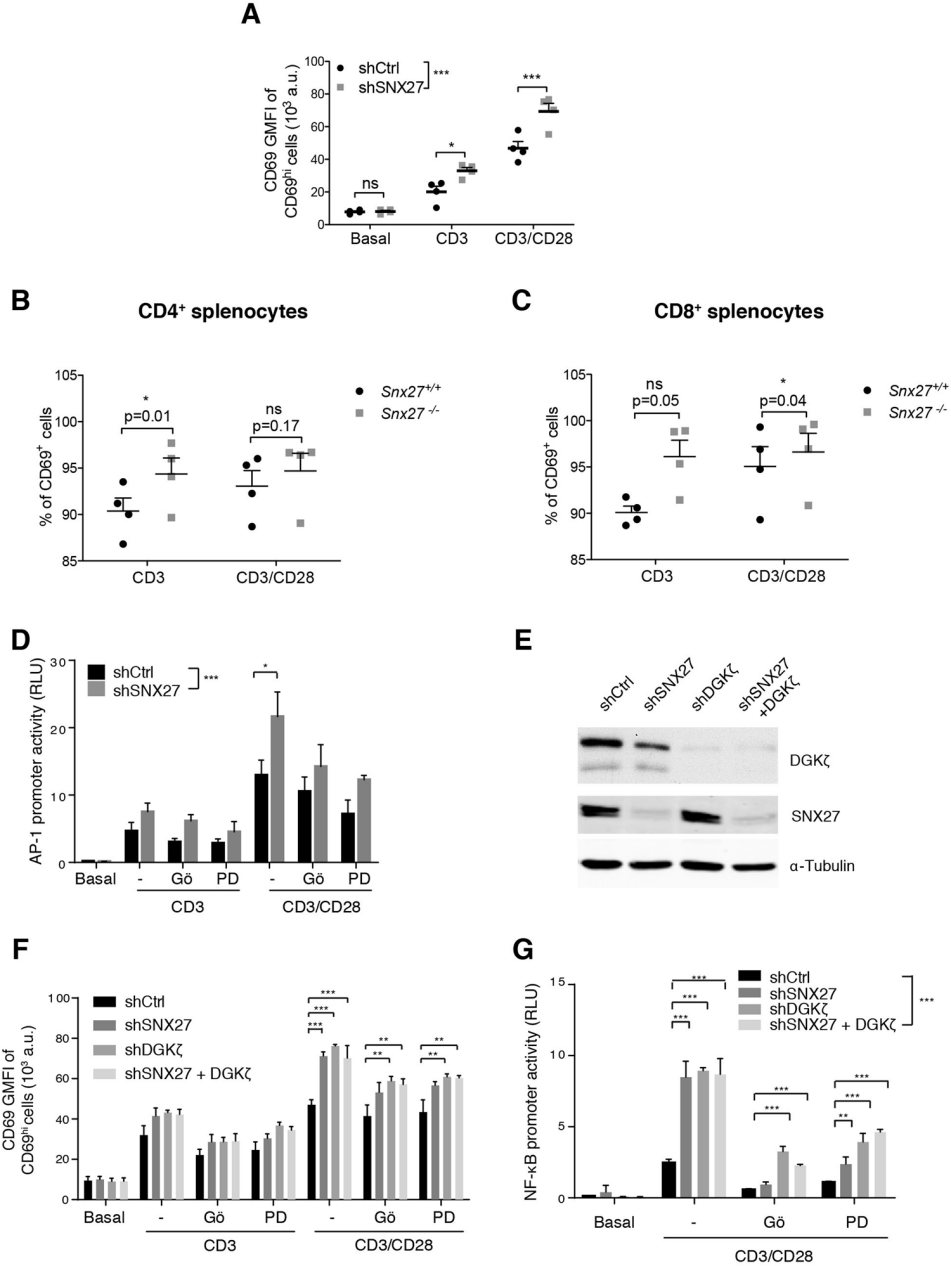
SNX27 limits activation of the Ras/ERK/AP-1 and NF-κB pathways. (**A**) shRNA-transfected Jurkat T cells were stimulated (6 h) with soluble anti-CD3 alone or with anti-CD28 for costimulation. Cells were stained for CD69 surface marker and, using flow cytometry, CD69^hi^ cells were gated, and geometric mean fluorescence intensity (GMFI) of CD69^hi^ cells was calculated. (**B**,**C**) Splenocytes from WT and *Snx27* ^−/−^ littermate pairs were stimulated (48 h) with plate-bound anti-CD3 alone or with soluble anti-CD28 for costimulation and stained for CD69 surface marker. The percentage of CD69^+^ cells were gated in CD4^+^ (**B**) or CD8^+^ (**C**) cells. Data are shown as mean ± SEM (ns no significant, p > 0.05; *p < 0.05; paired t-test; *n* = 4). Luciferase assays were performed to calculate (**D**) AP-1 and (**G**) NF-κB promoter activity. RLU, relative luciferase units (see Methods). Where indicated, cells were pretreated with Gö6976 or PD98059 for classic PKC or MEK inhibition, respectively. (**E**) Western blot analysis of cell lysates from shControl and shSNX27/DGKζ Jurkat T cells (**F**) shControl and shSNX27/DGKζ Jurkat T cells were treated as in A with or without inhibitors where indicated. Two-way ANOVA with the Bonferroni post-hoc test was used for multiple comparisons, both with GraphPad Prism 5 software. (ns) when p > 0.05, (*) when p < 0.05, (**) when p < 0.01, (***) when p < 0.001.

In the Jurkat T cells model, DGKζ silencing promotes PKCα/RasGRP1 activation that, upon antigenic triggering, mediates upregulation of the Ras/ERK axis^13^. We investigated the effect of SNX27 in the magnitude of Ras activation following T cell receptor (TCR) triggering. Activation of the Ras pathway can be monitored by measuring surface abundance of CD69, a C-type lectin that is transcriptionally upregulated in response to Ras/ERK activation after TCR stimulation^21^. SNX27-silenced Jurkat cells showed a significantly higher increase in the geometric mean fluorescence intensity (GMFI) of the CD69^hi^ population after T cell stimulation and costimulation (Fig. 1A).

Enhanced Ras/ERK signalling in DGKζ deficient T cells results in a greater proportion of CD69 expressing cells and prolonged expression of this activation marker^22^. When compared to WT mice, the analysis of long term CD69 induction in *Snx27* ^−/−^ T cells demonstrated a similar effect with higher percentages of CD69^+^ cells both in the CD4 (Fig. 1B) and CD8 populations (Fig. 1C). This is reminiscent of the larger proportion of CD69^+^ T cells observed at all antigen concentrations after stimulation of *Dgkz*ζ^−/−^ mouse T cells^22^, and suggests that SNX27, as DGKζ, also limits the Ras/ERK signaling threshold for activation in primary mouse T cells. The GMFI of the CD69^+^ cells was similar in WT and *Snx27* ^−/−^ cells (not shown), again resembling that described for ERK phosphorylation in *Dgkz* ^−/−^ mice^23^.

CD69 upregulation is largely due to AP-1 mediated transcription in response to activation of the Ras/ERK pathway. Analysis of the promoter activity of the AP-1-binding site in the CD69 promoter showed higher activity in SNX27-silenced compared to control Jurkat cells; CD28 costimulation markedly augmented this difference (Fig. 1D), which correlated well with the effect observed on CD69 induction in Jurkat and mouse T cells. To discriminate between inputs operating on ERK to CD69 transcriptional upregulation as the result of SNX27 silencing, cells were pretreated with the classic PKC (cPKC)-selective inhibitor Gö6976 (Gö) or PD98059 for MEK (MAPK/ERK kinase) before stimulation. In all cases, SNX27 depletion slightly increased AP-1 transcription independently of pharmacological inhibition of PKCα or MEK that decreased AP-1 dependent transcription to a similar extent in SNX27-silenced cells and controls (Fig. 1D).

Similar to that described for DGKζ deficiency, SNX27-silenced Jurkat T cells and SNX27-deficient splenocytes showed hyperactivation of DAG/PKC/Ras/ERK pathway, determined by the increased expression of the CD69 activation marker. To examine in more detail DGKζ contribution in SNX27-silenced cells, we compared CD69 expression in cells independently silenced for either SNX27 or DGKζ with cells silenced for the two proteins (Fig. 1E). CD69 induction upon CD3 or CD3/CD28 stimulation increased to a similar extent in double silenced cells when compared to silencing of SNX27 or DGKζ individually (Fig. 1F). The combined down-modulation of the two proteins did not have any appreciable additional effect in the presence of inhibitors (Fig. 1F).

In addition to PKCα dependent activation of the RasGRP1/Ras/ERK/AP-1 pathways, DGKζ silencing also enhances PKCθ activity downstream of CD28^19^. PKCθ triggering not only promotes ERK-dependent AP-1 transcription, but also activates the NF-κB-mediated transcription program^15^. Analyses of the promoter activity of the NF-κB binding site in the CD69 promoter confirmed increased activation in SNX27-silenced cells that paralleled the one observed upon DGKζ silencing (Fig. 1G). Dual silencing of the two proteins did not induced additional effects, supporting the idea that down-regulation of NF-κB activity by DGKζ is dependent on SNX27 expression.

### SNX27 regulates the PKCα/DGKζ axis

**Figure 2:**
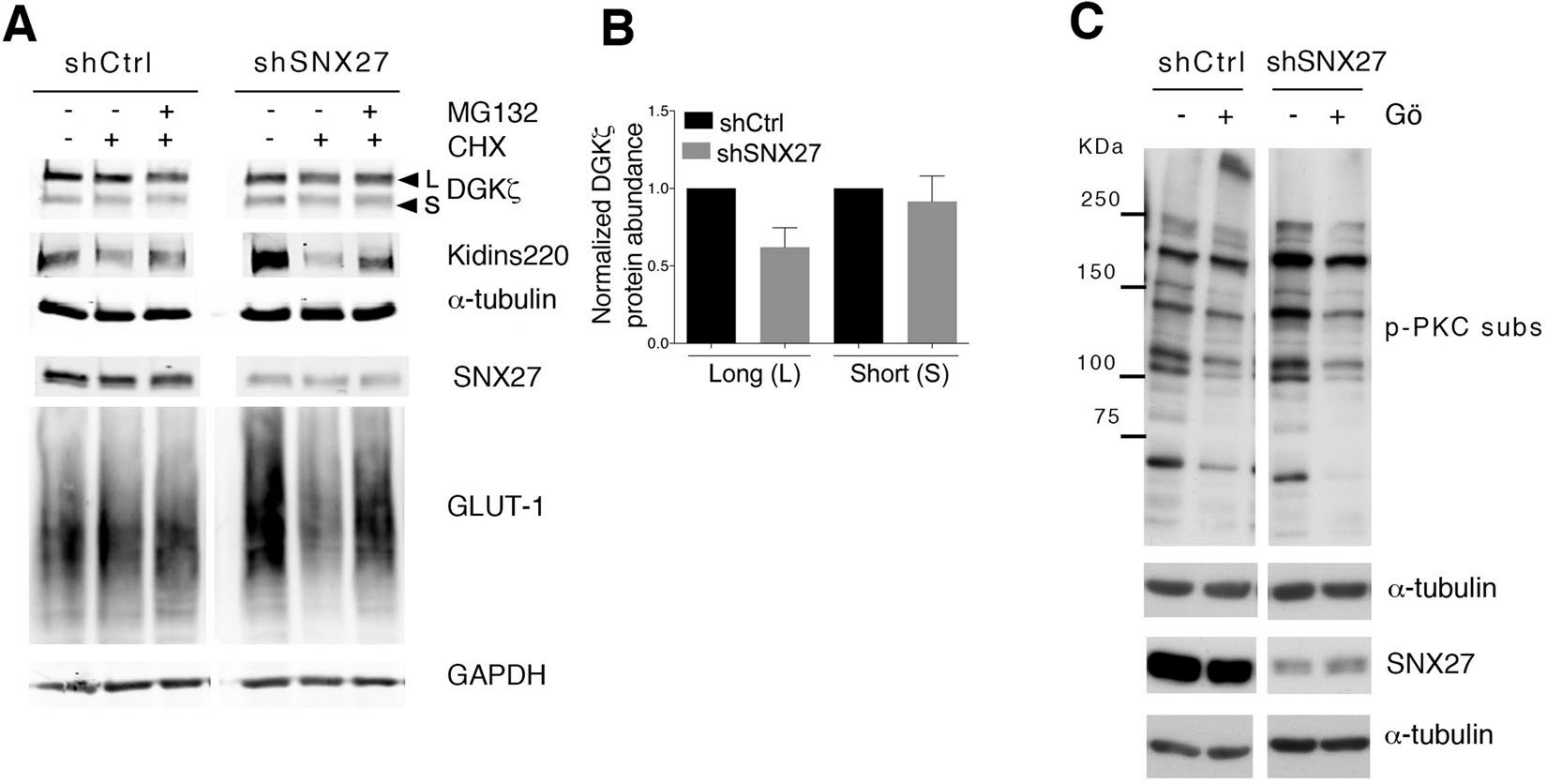
SNX27 silencing triggers PKC activation in basal conditions. (**A**) shControl and shSNX27 Jurkat T cells were treated with the indicated inhibitors (10 μg/ml cycloheximide (CHX); 5μM MG-132; 100 nM Gö) for 6 h. Total levels of the indicated proteins were measured by western blot. After transfer blots were excised at the approapiate Mw and probed for detection of the indicated proteins. Results are shown from a representative experiment of at least three independent experiments that rendered similar results. (**B**) Quantification of DGKζ (top) long and (bottom) short splicing isoforms levels 72-96 h after shRNA transfection. Data shown as mean ± SEM from at least three independent experiments. (**C**) shControl and shSNX27 Jurkat T cells were left untreated or treated with Gö for 6 h. Blots were incubated with an antibody that detects PKC phosphorylated substrates. Duplicate samples were analysed for SNX27 silencing. Representative experiment of three independent experiments that rendered similar results.

The best-known function of SNX27 interaction with its transmembrane cargoes is to facilitate their recycling to the cell surface, protecting them from degradation^5^. As a result, SNX27 silencing is reported to accelerate lysosomal dependent degradation of its partners. We reasoned that the similar effects observed upon SNX27 or DGKζ silencing could be interpreted as the result of DGKζ degradation. We thus examined DGKζ stability in SNX27 silenced Jurkat T cells. As a control, we monitored the abundance of the transmembrane proteins GLUT1 and Kidins220, both known transmembrane SNX27 PDZ cargoes^5^. Western blot analyses of DGKζ in SNX27-silenced Jurkat T cells showed no substantial changes in the protein levels of the short isoform and only modest variations in the abundance of the long isoform (Fig. 2A,B). SNX27 silencing revealed increased levels of Kidins220 and GLUT-1 (Fig. 2A). To assess if increased protein expression was the result of protein synthesis we examined the effect of inhibition of protein synthesis by cycloheximide (CHX). CHX treatment had no obvious effect in control cells but acutely decreased GLUT1 and Kidins220 abundance in SNX27-silenced cells (Fig. 2A), suggesting that impaired recycling of SNX27 cargoes in Jurkat T cells is compensated by enhanced protein synthesis. Diminished GLUT1 and Kidins220 abundance in CHX treated cells was not recovered by inhibitors of the lysosome pathway (not shown) and partially recovered by proteasome inhibition. DGKζ did not behave as one of these classical SNX27 PDZ cargoes, suggesting that DGKζ stability is independent of SNX27 interaction. The best-characterized DGKζ function is that of restricting PKC activity through DAG consumption^19^. The impact of SNX27 silencing on basal PKC activity was assayed using an antibody that recognizes phosphorylated PKC substrates. Basal phosphorylation of PKC substrates increased upon SNX27 silencing, and was sensitive to Gö6976 (Gö) (Fig. 2C) suggesting increased activity of cPKCs in the absence of SNX27.

### Analysis of SNX27 deficiency on T cell development

The previous experiments indicated that, in the absence of SNX27, DGKζ cannot fulfill its role as a negative regulator of DAG-triggered signals. DGKζ-deficient mice show peripheral T cells with hyperactive responses to TCR stimulation but normal T cell development^22^. To further examine SNX27 contribution to the regulation of DGKζ functions, we characterized T cell development in SNX27 knockout mice (*Snx27* ^−/−^). *Snx27* ^−/−^ mice were originally reported to die at 4 weeks post-partum due to postnatal growth defects^24^. Optimization of housing and feeding conditions allowed us to analyze mice from 6 to 12 weeks old. Crossing *Snx27* ^+/−^ heterozygotes on C57BL/6 and 129SV mixed backgrounds^24^ generated F1 hybrid background *Snx27* ^+/+^ and *Snx27* ^−/−^ mice and enabled paired analysis of littermates.

**Figure 3:**
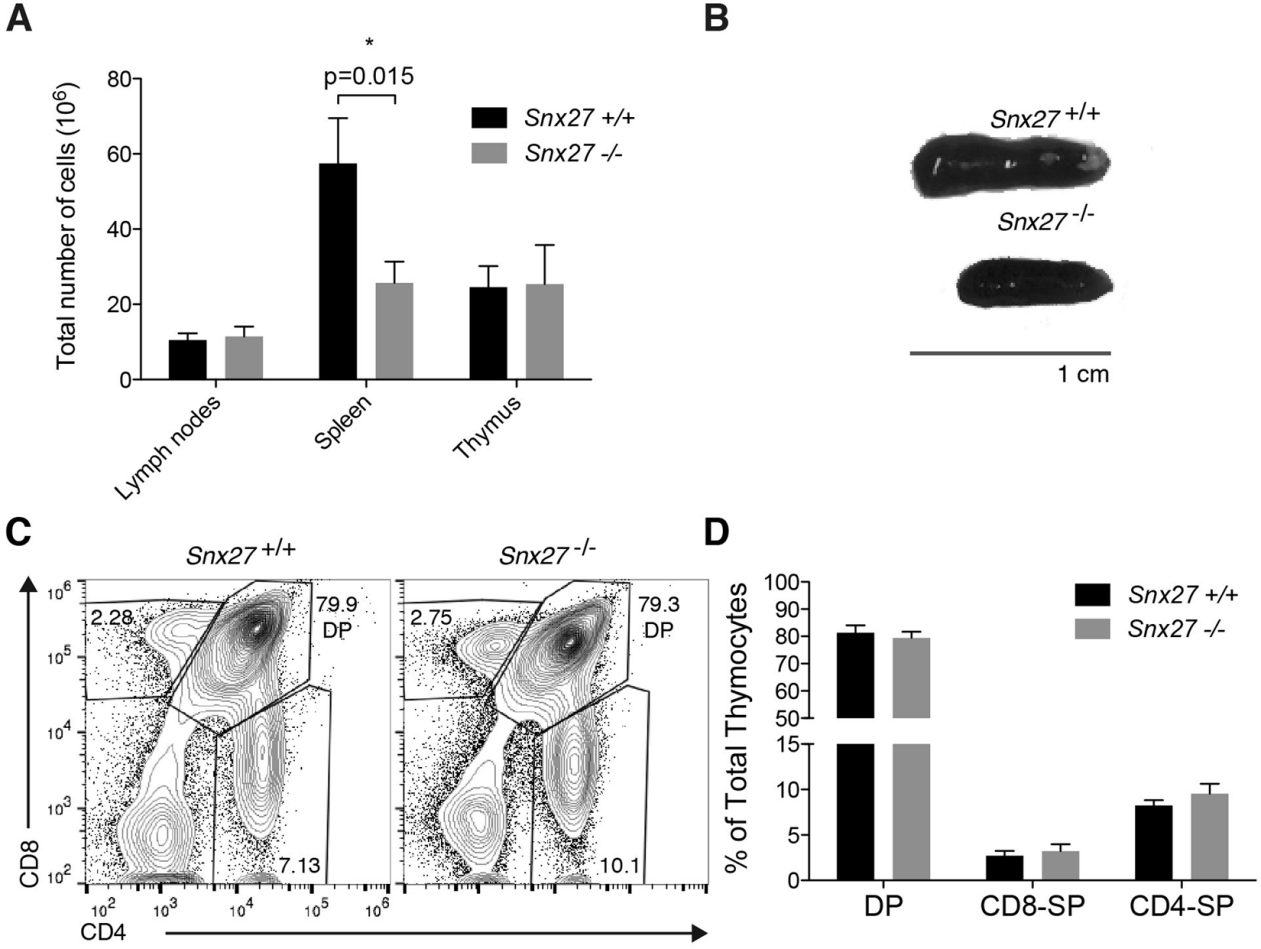
Analysis of T cell development in WT and *Snx27* ^−/−^ mice. (**A**) WT and *Snx27* ^−/−^ mice littermate-matched pairs of mice were sacrificed and total cellularity recorded of the thymus, spleen and lymph nodes. Data shown as mean ± SEM (*p < 0.05; paired t-test; *n = *7). (**B**) Spleen from WT and *Snx27* ^−/−^ mice littermate-matched pairs are shown for size comparation. (**C**,**D**) Thymocytes from WT and *Snx27* ^−/−^ littermates pairs were stained for CD4 and CD8 and analyzed by flow cytometry. (**C**) Representative flow cytometry plots. (**D**) The percentage of total double-positive (DP, CD4+ CD8+) and single positive (SP, CD4+ CD8− or CD4−CD8+) thymocytes was calculated. Data shown as mean ± SEM (*p < 0.05; paired t-test; n = 7).

Analysis of total cellularity of the lymphoid organs: thymus, spleen and lymph nodes (LN) in *Snx27* ^−/−^ mice showed no defect in the appearance or cellularity of thymus and LN (Fig. 3A). Consistent with previous reports showing that *Snx27* deletion in mice results in smaller animals with reduced organ size^24^, *Snx27* ^−/−^ mice showed a significant decrease in spleen size (Fig. 3B) that correlated with reduced cellularity compared to wild type (WT) littermate controls (Fig. 3A). In the thymus, thymocytes develop from the CD4^−^CD8^−^ double negative cell population (DN) to the CD4^+^CD8^+^ double positive (DP) stage and then to the single positive (SP) CD4^+^ and CD8^+^ mature T cells. To evaluate the development of *Snx27* ^−/−^ T cells, we stained thymocytes for cell surface expression of CD4 and CD8 and analyzed the different populations by flow cytometry. As described for DGKζ deficient mice^22^, the analysis of DP and SP thymocyte populations showed no significant differences (Fig. 3C,D).

### Analysis of SNX27 deficiency on T cell homing

**Figure 4:**
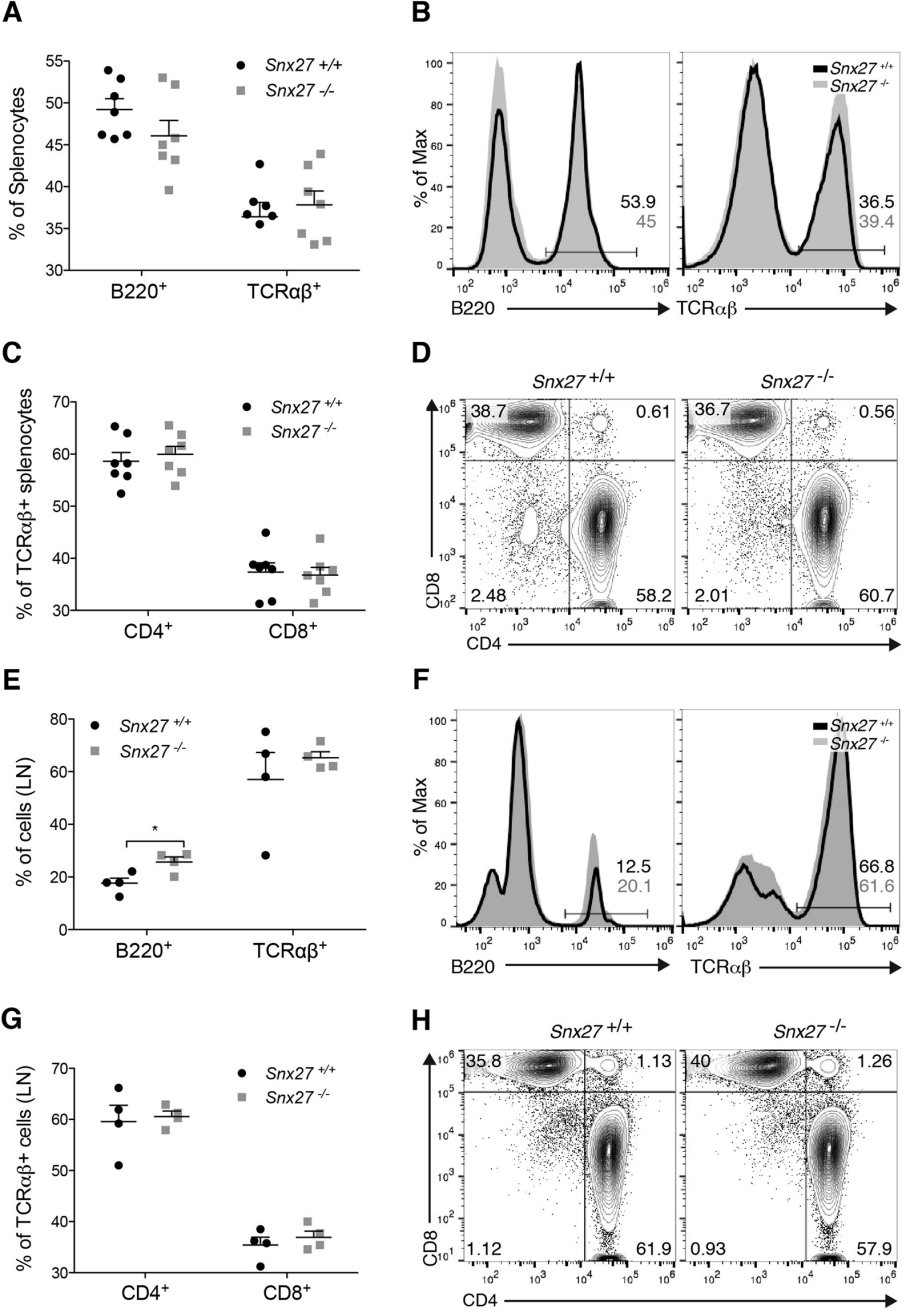
*Snx27* ^−/−^ mice show normal peripheral T cell populations. (**A**–**D**) Splenocytes and (**E**–**H**) lymph node T cells from WT and *Snx27* ^−/−^ littermate pairs were stained for the indicated cell surface markers and analyzed by flow cytometry. (A,B,E,F) panels show percentage of B220+ and TCRαβ+ cells. In (**C**,**D**,**G** and **H**) percentages of CD4+ and CD8+ cells were measured in the TCRαβ+ population. (B,D,F,H) panels show representative plots. (A,C,E and G) panels show total data presented as mean ± <SEM (*p < 0.05; paired t-test; A, C n = 7; E, G n = 4).

Mature T lymphocytes exit the thymus and migrate to peripheral lymphoid organs where mature B cells also reside^25^. Albeit the spleen showed reduced cellularity, in agreement with its reduced size, quantification did not reveal major differences in the percentages of B and T cell populations in spleen (Fig. 4A,B) nor lymph nodes (Fig. 4E,F). *Snx27* ^−/−^ mice showed no gross differences with WT littermates in the percentages of CD4 and CD8 cells gated in TCRαβ^+^ (Fig. 4C,D,G,H). Overall the results indicate that *Snx27* deletion in mice, as that of *Dgkz*, did not markedly alter thymic T cell development. In spite of their reduced size, *Snx27* ^−/−^ mice nonetheless maintained peripheral T cell populations similar to WT littermates.

### SNX27 deficiency promotes PKC-regulated functions but impairs activation of the AKT/mTOR axis

**Figure 5:**
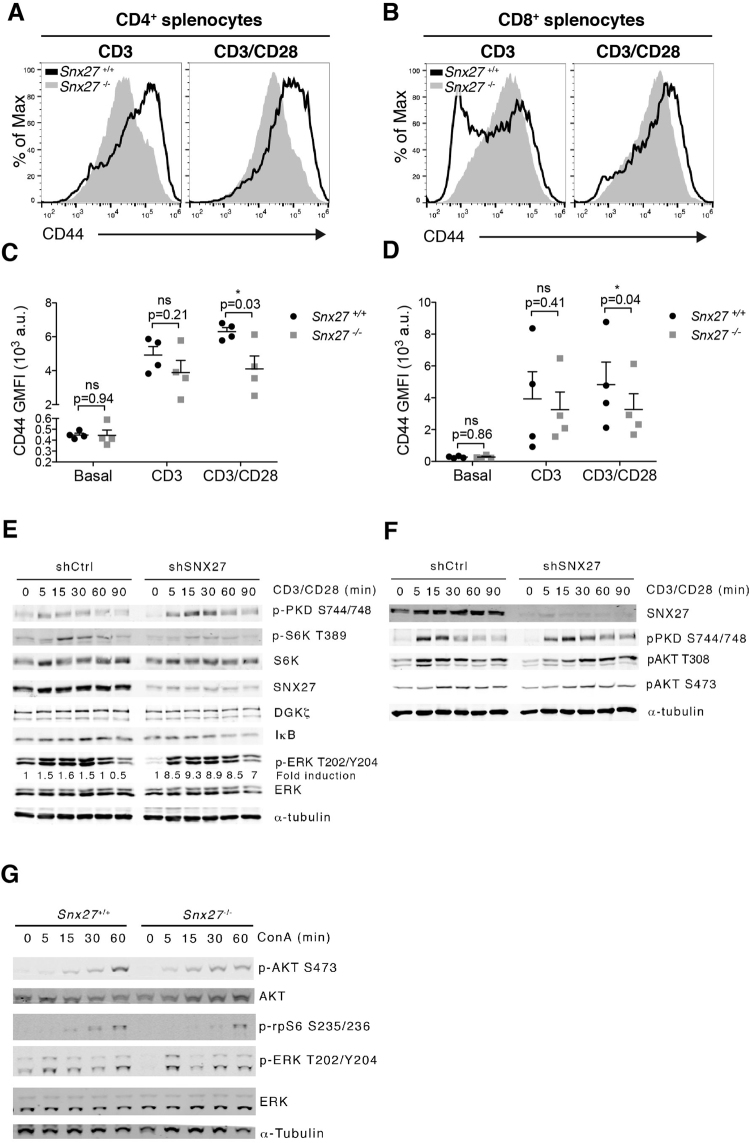
Effect of SNX27 silencing on TCR/CD28 triggered signals. (**A**,**B**) Splenocytes from WT and *Snx27* ^−/−^ littermate pairs were stimulated (48 h) with plate-bound anti-CD3 alone or with soluble anti-CD28 for costimulation and stained for CD44 surface marker. Representative flow cytometry plots for CD44 induction are shown. (**C**,**D**) CD44 GMFI were calculated. Data are shown as mean ± SEM (ns no significant, p > 0.05; *p < 0.05; paired t-test; *n* = 4) a.u., arbitrary units. (**E**,**F**) Control and SNX27-silenced cells were stimulated with anti-CD3/anti-CD28 antibodies for the indicated times. Western blot analysis of cell lysates showed phospho- and total protein abundance of the indicated proteins. Phospho-ERK signals were normalized to total ERK abundance, and results for each time point were also normalized to unstimulated cells to calculate fold induction. Blots are representative experiments from three independent experiments that provided similar results. (**G**) Splenocytes from WT and *Snx27* ^−/−^ littermate pair were stimulated at the indicated times with concanavalin A (ConA). Phosphorylation of the indicated proteins was evaluated by western blot in total cell lysates. Tubulin was used as a loading control. A representative experiment from three independent ones is shown.

CD69 upregulation is a very sensitive but transient measure of antigen recognition. Expression of CD44 is also induced after antigen recognition, and remains high on all antigen-experienced cells^26^. At difference from that observed in WT splenocytes, the analysis of the CD44^+^ T cell population in *Snx27* ^−/−^ cells revealed no substantial increase following CD3/CD28 costimulation (Fig. 5A and B). The quantification of paired littermates showed significantly lower GFMI in CD4 T cells (Fig. 5C). CD44 GMFI was slightly reduced in *Snx27* ^−/−^ activated CD8 T cells in response to either anti-CD3 or CD3/CD28 costimulation (Fig. 5D).

The analysis of CD69 induction correlated with *Snx27* ^−/−^ mice displaying a larger population of responsive T cells, whereas low cell surface CD44 abundance suggested impairment in the ability to achieve a full activation phenotype. To better clarify the contribution of SNX27 to the correct activation of signals, we made use of the Jurkat T cell model. Analysis of SNX27 silenced cells revealed enhanced PKD phosphorylation, a dual, direct and PKCθ-mediated DAG effector that is very sensitive to DGKζ silencing^19^ (Fig. 5E). Enhanced PKCθ activation as the result of DGKζ deficiency also promotes TCR/CD28 dependent phosphorylation and degradation of the NF-κB inhibitor IκB^19^. In accordance, SNX27 silencing led to increased IκB degradation compared to controls (Fig. 5E).

CD28 engagement also activates phosphatidylinositol (PtdIns) 3-kinase (PI3K), that favours PtdIns-dependent kinase 1 (PDK-1) and AKT recruitment and activation. This pathway activates the metabolic regulator mammalian target of rapamycin (mTOR)/S6 kinase (S6K) pathway (reviewed in^27,28^). DGKζ silencing facilitates the association of PKCθ and PDK1 and enhances mTOR/S6K pathway activation downstream CD28^19^. In contrast with PKCθ enhanced functions, SNX27-silenced cells showed reduced mTOR activation determined by S6K phosphorylation at its Thr389 residue (Fig. 5E). Analysis of AKT confirmed a decrease in the initial phosphorylation at Ser 308, the PDK-1 phosphorylation site (Fig. 5F). In agreement with the data from SNX27 silenced Jurkat T cells, phosphorylation of the S6K effector rps6 (ribosomal protein S6) at Ser235/6 was delayed in *Snx27* ^−/−^ splenocytes (Fig. 5G). AKT Ser473 phosphorylation was also reduced in *Snx27* ^−/−^ splenocytes at later times, which indicated alterations in mTORC2 pathway downstream of TCR triggering.

### Differential effect of SNX27 and DGKζ silencing on IL-2 production

**Figure 6:**
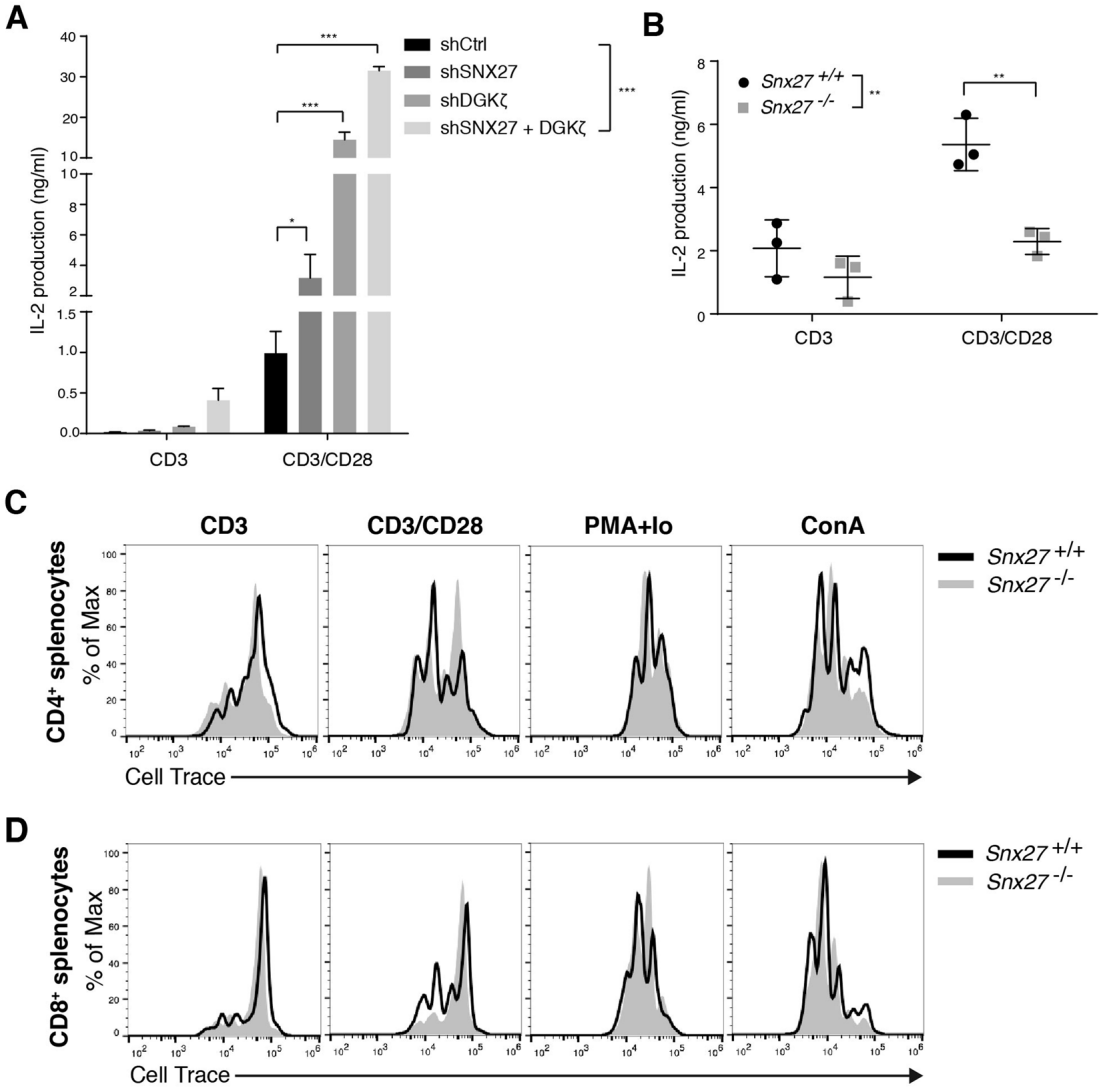
Effect of SNX27 silencing on IL-2 production and T cell proliferation. (**A**) shRNA-transfected cells were stimulated (16 h) with soluble anti-CD3 alone or with anti-CD28 and IL-2 concentration in the supernatant was measured by ELISA. Data shown as mean ± SEM (ns, not significant, p > 0.05; *p < 0.05; **p < 0.01; ***p < 0.001; two-way ANOVA/Bonferroni post test; *n* = 4). (**B**) Splenocytes from WT and *Snx27* ^−/−^ littermate pairs were stimulated (48 h) with plate-bound anti-CD3 alone or with soluble anti-CD28 and IL-2 concentration in the supernatant was measured by ELISA. Data shown as mean ± SEM; (**) p < 0.01, paired t-test *n* = 3). (**C**,**D**) Splenocytes from WT and *Snx27* ^−/−^ littermate pairs were labeled with Cell Trace Violet and stimulated (48 h) with plate-bound anti-CD3 alone or with soluble anti-CD28 and with PMA/ion or ConA. Cells were then stained for CD4 and CD8 surface markers and analyzed by flow cytometry. (**A**) CD4^+^ or (**B**) CD8^+^ cells were gated and flow cytometry plots of Cell Trace Violet intensity were analyzed. Data shown are representative of three independent experiments.

The analysis of SNX27 silenced Jurkat cells correlates with that observed in SNX27 deficient T cells and suggest that, differently from DGKζ, SNX27 is necessary for the correct activation of the AKT/mTOR/S6K pathway downstream of CD3/CD28 triggering. The integration of PKCθ and AKT signals downstream TCR and CD28 are indispensable to sustain IL-2 production^29^. The enhanced activation of PKCθ and AKT pathways in DGKζ null cells heightens IL-2 production both in genetically modified^22^ and silenced activated T cells^19^. We next determined the effect of SNX27 silencing on IL-2 production and compared it with that observed upon DGKζ silencing. IL-2 production in Jurkat T cells was exclusively detected in the presence of costimulation (Fig. 6A). SNX27 silencing resulted in minor increase on IL-2 production in sharp contrast with the enhanced synthesis observed in DGKζ-silenced cells (Fig. 6A). Double silencing of DGKζ and SNX27 in Jurkat T cells resulted in further increase on IL-2 production upon costimulation (Fig. 6A). These results suggest that, in contrast to their partnership in DAG-dependent transcriptional control, DGKζ limits IL-2 production independently of SNX27 interaction. A similar analyses of IL-2 production in SNX27-deficient T cells showed diminished IL-2 production upon activation (Fig. 6B), confirming a differential effect with that described for DGKζ deficient mice.

Recognition of antigens by naïve T lymphocytes triggers an activation program that induces growth and ultimately cell division. Enhanced IL-2 production in DGKζ deficient T cells correlates with hyperproliferation of naïve T cells in response to antigenic challenge^22^. We monitored cell proliferation in SNX27-defficient T cells. Before stimulation, WT and *Snx27* ^−/−^ splenocytes were stained with CellTrace and at 48 h after stimulation, stained for CD4 and CD8 expression and analysed by flow cytometry. Proliferating cells were identified by dye dilution. Compared to WT littermates, CD4 and CD8 populations from *Snx27* ^−/−^ mice proliferated normally in response to CD3 stimulation and showed a slight delay in their division upon co-stimulation (Fig. 6C,D). To determine whether *Snx27* deficiency induced some intrinsic defect that prevented a high division index, we used pharmacological, more potent proliferative stimuli. Cell treatments with either phorbol 12-myristate 13-acetate (PMA) plus ionomycin or concavalin A (Con A) resulted in similar numbers of dividing cells in WT and *Snx27* ^−/−^ T cells (Fig. 6C and D).

### SNX27 silencing limits Transferrin receptor expression

**Figure 7:**
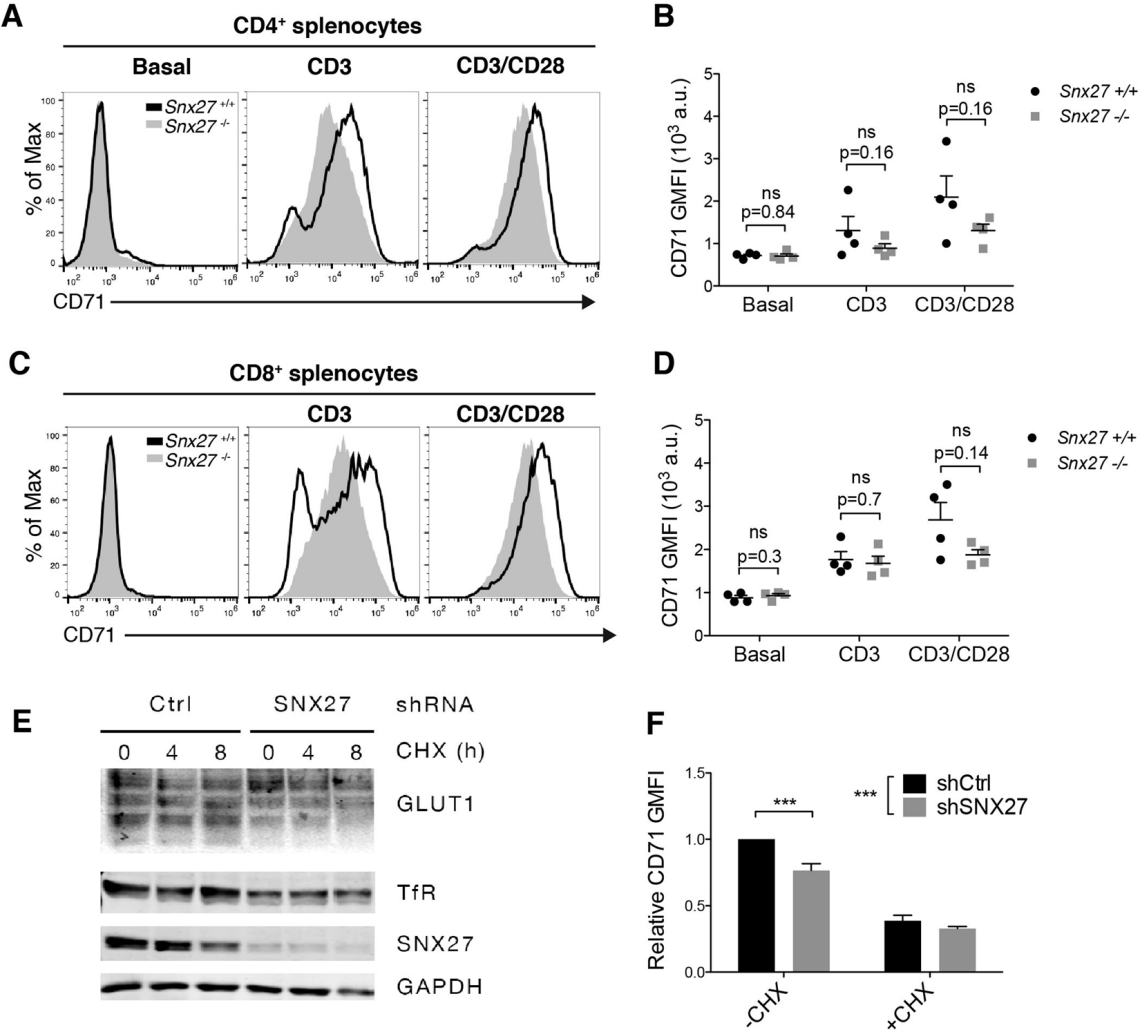
Splenocytes from WT and *Snx27* ^−/−^ littermate pairs were stimulated (48 h) with plate-bound anti-CD3 alone or with soluble anti-CD28 and stained for CD71. Using flow cytometry, CD4^+^ and CD8^+^ cells were gated. (**A**,**C**) Representative flow cytometry plots are shown. (**B**,**D**) CD71 GMFI was calculated. Data shown as mean ± SEM; (ns: no significant, p 0.05, paired t-test *n* = 4). (**E**) shControl and shSNX27 Jurkat T cells were treated with 10 μg/ml cycloheximide (CHX) for the times indicated and total levels of GLUT1, TfR, SNX27 and GAPDH as loading control were determined by western blot. Graph represents a typical experiment from three independent experiments repeated with similar results (**F**) shControl and shSNX27 Jurkat T cells were treated with 10 μg/ml cycloheximide (CHX) for 4 h and cell surface levels of TfR(CD71) were determined by flow cytometry. Geometric mean fluorescence intensity (GMFI) was normalized to untreated shControl cells. Data shown as mean mean ± SEM (**p < 0.01; ***p < 0.001; two-way ANOVA/Bonferroni post test) from at least three independent experiments.

Our experiments suggested that impaired activation of the AKT/mTOR/S6K pathway in *Snx27* ^−/−^ T lymphocytes did not grossly affected cell proliferation. In mature peripheral T cells the extension of activation of the AKT/mTOR/S6K axis controls protein synthesis, nutrient uptake and cell growth of antigen-activated T cells^30,31^. Optimal growth responses in lymphocytes are associated with the upregulation of transferrin receptors that deliver iron, a necessary co-factor for several metabolic reactions, to the cell^32^. The levels of expression of CD71 (the transferrin receptor; TfR) is markedly upregulated upon costimulation and reflect the amplitude of mTOR activation^32^. Analysis of CD71 surface expression in WT splenocytes confirmed enhanced expression following costimulation. *Snx27* ^−/−^ T cells upregulated CD71, although it was less abundant than in WT controls (Fig. 7A–D).

Jurkat T cells constitutively express TfR so we determined if SNX27 silencing had any effect on receptor expression. Western blot analysis of SNX27-silenced Jurkat T cells showed decreased TfR expression. In contrast to that observed for GLUT1, TfR abundance remained constant after CHX treatment, suggesting a defect independent on receptor recycling (Fig. 7E). Flow cytometry analysis confirmed reduced TfR/CD71 cell surface expression after SNX27 silencing with no significant differences between control and SNX27-silenced cells after CHX treatment (Fig. 7F).

### SNX27 is required for TCR/CD28-dependent induction of T cell growth

**Figure 8:**
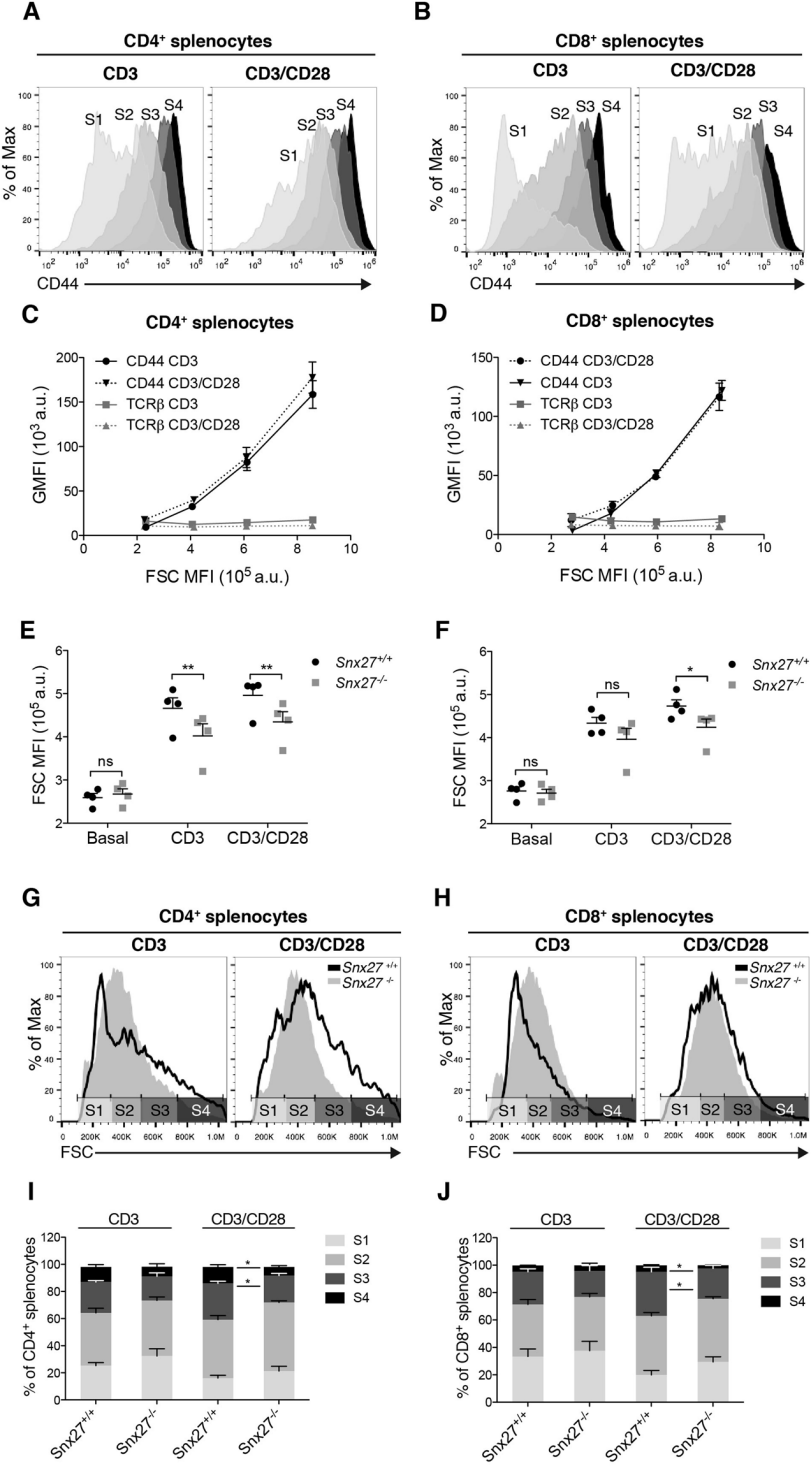
*Snx27* deficient splenocytes show reduced growth upon costimulation. Splenocytes from WT and *Snx27* ^−/−^ mice were stimulated for 48 h with plate-bound anti-CD3 alone or in combination with soluble anti-CD28 and stained for the indicated markers. Using flow cytometry, CD4^+^ and CD8^+^ cells were gated. (**A**,**B**) Representative flow cytometry plots showing CD44 expression in the four populations selected by size (S1, S2, S3, S4) (**C**,**D**) GMFI of CD44 (black) and TCRβ (grey) were calculated in cells from the four distinctly sized populations (Data shown as mean ± SEM *n* = 2). (**E**,**F**) Splenocytes from WT and *Snx27* ^−/−^ littermate were stimulated as in A and cell size of CD4^+^ and CD8^+^ populations was calculated, (**G**,**H**) representative flow cytometry plots of stimulated cells, (**I**,**J**) percentage of cells in the four distinctly sized populations. a.u., arbitrary units.

In recently activated T lymphocytes, cell size can be used as a surrogate indicator of mTOR activity^33^. In cancer T cells, expression of CD44 has been linked to enhanced mTOR activation as the result of RasGRP1 mutations^34^. We examined cell surface CD44 abundance relative to T cell size following stimulation. We calculated the CD44 GMFI of cells from four distinctly sized populations [from the smallest FSC MFI population (“size 1”; S1) to the largest (“size 4”; S4) (Fig. 8A,B), and used cell surface TCRβ expression as a control. Our results showed a constant value for TCRβ GMFI and isotype controls independently of cell size whereas CD44 GMFI was directly proportional to FSC MFI (Fig. 8C,D). As a consequence, the S3 and S4 populations in CD4 and CD8 cells expressed the highest CD44 levels (Fig. 8A and B).

These data confirm that CD44 expression in recently activated T cells is a direct reporter of cell growth, and indicate that the defective expression of CD44 in *Snx27* ^−/−^ T cells could be linked to impaired mTOR activation and concomitant cell growth. We next measured cell size in WT and *Snx27* ^−/−^ T cells before and after activation. Naïve *Snx27* ^−/−^ T cells were the same size as WT T cells (Fig. 8E,F), but they had reduced size after CD3 or CD3/CD28 stimulation. Analysis of cell distribution among distinctly sized populations (gates shown in Fig. 8G,H), demonstrated that CD3-stimulated T cells were found mostly in the smaller S1 and S2 populations, with no gross differences between WT and *Snx27* ^−/−^ T cells. CD28 costimulation resulted in a marked increase of the percentage of WT T cells distributed in the larger sized populations (S3 and S4), whereas the percentage of these populations in CD4^+^ and CD8^+^ *Snx27* ^−/−^ cells remained low (Fig. 8I,J). These results correlate with previous findings and suggest that *Snx27* ^−/−^ naïve T cells present a growth defect upon strong antigenic challenge.

## Discussion

In this study we have explored in more depth the contribution of SNX27 to the regulation of the signals triggered by antigen recognition. Our results confirm the relevance of SNX27 interaction with DGKζ by showing that DGKζ’s role as a negative regulator of DAG signals requires SNX27. We demonstrate that the effect of SNX27 silencing generally mirrors that of DGKζ depletion, suggesting that SNX27 contributes to sustain the active pool of DGKζ. SNX27 silencing impairs DGKζ function without grossly affecting protein levels, suggesting a failure for correct localization and/or function.

Our studies showing basal PKC activation in SNX27 silenced cells, suggest a failure in the crosstalk between DGKζ and PKCα. These two proteins exist in a tight equilibrium where local DAG regulation by DGKζ limits PKCα activation, whereas upon activation PKCα phosphorylates DGKζ disrupting their interaction^35,36^. Studies in other systems have suggested that the crosstalk between DGKζ and PKCα requires PDZ containing scaffolds. At neuronal synapses, the PDZ-containing scaffold protein PSD-95 (postsynaptic density protein) enables this regulatory mechanism in basal conditions^37^. In neuromuscular junctions, the PDZ proteins syntrophins facilitate PKC-dependent localization of DGKζ to junctions^38^. In T cells, where reciprocal PKCα/DGKζ modulation also occurs^13^, SNX27 acts as an endosomal scaffold for DGKζ to control basal PKC activity. We hypothesize that PKC activation, as the result of the disruption of SNX27/DGKζ association, reflects impaired DAG consumption at the endosomal/recycling compartment where SNX27 resides.

Antigenic recognition by T cells triggers rapid elevation of DAG at the contact area. The rapid recycling of DAG-enriched organelles together with local generation of DAG by PLCγ– dependent PIP2 hydrolysis helps to sustain activation of DAG effectors at this particular localization. Functional inactivation of DGKζ in recycling endosomes as a result of SNX27 silencing correlates with the enhanced activation of DAG-regulated pathways triggered by co-stimulatory signals. SNX27-silenced cells showed enhanced activation of DGKζ-regulated pathways including enhanced NF-κB and AP-1 promoter activity upon costimulation. The effects of silencing SNX27 and DGKζ on CD69 upregulation and NF-κB activity were not enhanced in double-silenced T cells, which supports the hypothesis that SNX27 controls DAG signaling through DGKζ interaction. The analysis of *Snx* *27* ^−/−^ mice demonstrated normal thymus development and hyperactivation of DAG-mediated signals in mature T cells, similar to that described for dgkζ^−/−^ mice. Consistent with previous reports^24^ *Snx27* ^−/−^ mice showed a significant decrease in spleen size in agreement with the reduced size of the animals. Reduced spleen size correlated with small number of cells although the percentage and size of T cells was maintained. In agreement with that observed in Jurkat T cells, *Snx27* ^−/−^ splenocytes revealed normal activation of the Ras/ERK pathway and enhanced expression of the CD69 activation marker.

DAG regulated transcription is essential for T cells to synthesize and secrete IL-2. In contrast to the non-additive effects on NF-κB and AP-1 promoter activity observed in the luciferase reporter assays, T cells silenced for both DGKζ and SNX27 showed enhanced IL-2 production compared to those silenced for SNX27 alone. This suggests that not all of the functions attributed to DGKζ are impaired following SNX27 silencing. In agreement, SNX27 silencing partly reduced S6K activation in CD3/CD28 activated T cells, while previous reports found that DGKζ silencing led to increased CD3/CD28-dependent activation of the mTOR/S6K pathway^19^.

Impaired mTOR activation correlated with defective growth of naïve T cells in response to antiCD3/CD28 stimulation. mTOR is a major regulator of T cell metabolism and its activation promotes the expression of nutrient receptors like CD71 required to support cell growth^27^. Our studies agree with those showing that T cell size after TCR/CD28 costimulation correlates with mTOR activity^33^ and demonstrate that CD44 expression, a reporter of tonic mTOR activity in T cells^34^, is also a marker of T cell growth upon activation. mTOR is a nutrient sensor activated by amino acids and glucose that, in T cells is tightly regulated by activation of the PI3K/PDK-1/AKT axis. DGKζ-silenced T cells and DGKζ deficient mice show enhanced AKT and mTOR dependent S6K activation^19^. Ectopic DGKζ overexpression impairs mTOR activation in a kinase dependent manner^39^. SNX27 silencing does not affect DGKζ expression, suggesting that DGKζ inhibitory function on the AKT/mTOR axis is enhanced in the absence of SNX27. In this regard, double SNX27 and DGKζ silencing in Jurkat T cell enhances IL-2 production. Additional SNX27 cargoes may also contribute to the differential effects observed for TCR-mediated AKT/mTOR activation. Proteomic studies and *in silico* analysis demonstrate that there are hundreds of potential proteins that can interact with SNX27^7^. DGKζ is a high affinity SNX27 interactor, so DGKζ silencing could favor SNX27 nteraction with other cargoes enhancing the regulation of the mTOR/S6K axis. SNX27 deficiency on the other hand would limit this function. Additional studies should explore in depth if DGKζ contributes to limit TCR-triggered mTOR activation in SNX27 deficient T cells.

SNX27-mediated control of mTOR correlates with other studies in mice where low or null expression of the proteins that participate in the mTOR signaling pathway results in small animals with reduced organ size (reviewed in^40^). *Snx27* ^−/−^ mice are indeed smaller than controls^24^. Nevertheless, while we observed decreased spleen cellularity in *Snx27* ^−/−^ animals compared to controls, thymus and LN cellularity were unaltered. Normal thymus and LN cellularity concur with no gross defects in T cell development. Thymic size and thymopoiesis capacity are determined by thymic niche availability and by T cell progenitor dosage, which depends on cell proliferation and apoptosis during development and thymic involution (reviewed in^41^). Additional studies are needed to fully explore if the lack of SNX27 affects hematopoietic cell development. It will also be of interest to examine if the minor size of SNX27 defficient mice is due to alterations in the correct activation of the AKT/mTOR/S6K pathway.

In summary our studies demonstrate that SNX27 interaction with DGKζ enables adequate metabolism of the DAG that is generated during T cell activation. In addition, SNX27 facilitates the normal growth of naïve, quiescent T cells when they have high metabolic demands. Albeit currently considered linked events, cell growth and cell cycle progression are nonetheless distinct processes in mammalian cells. Our studies corroborate this observation and agree with studies showing that, in T lymphocytes, mTOR promotes cell cycle progression but is not strictly necessary for proliferation (reviewed in^42^). *Snx27* ^−/−^cells, with a clear defect in cell growth, were indeed able to proliferate normally. Additional studies should explore if the severe growth limitations in *Snx27* ^−/−^ T cells that we describe here, could result in alterations in T cell differentiation and expansion of effector cell populations.

## Methods

### Antibodies and reagents

We used anti-CD3 and -CD28 monoclonal antibodies (555336, 555725, 553058, 553295; BD PharMingen) for T cell stimulation. For cytometry analysis, we used anti-human-CD69-PE, anti-mouse CD44-FITC (IM1943, 731957; Beckman Coulter), CD4-PECy5, CD8-PeCy7 (100434, 100722; Biolegend), CD69-FITC, CD71-PE (553236, 553267; Pharmingen) and the isotype control mouse IgG1-PE (556029; Pharmingen). For western blot, we used anti-pERK 1/2 (T202/Y204), -ERK 1/2, -pPKD S744/748, -pAKT T473, -Akt, -IκB, -pS6K (T389), -S6K, -prpS6 (S235/236), -pPan-PKC substrate (4370, 4696 S, 2054 L, 4060, 2910 S, 9242 S, 9206 L, 2708, 2211 S, 2261 L; Cell Signaling), -PKD, -GAPDH (sc-935, sc25778; Santa Cruz), anti-α-tubulin (9026; Sigma-Aldrich), -DGKζ, -SNX27, -GLUT1 (αβ105195, ab77799, ab15309; Abcam), anti-Kidins220 described in^43^ was a kind gift from Dr Teresa Iglesias. The following secondary antibodies were used: horseradish peroxidase (HRP)-conjugated anti-mouse and -rabbit IgG (P0447, P0448; Dako), anti-rabbit IgG Dylight 800 (SA5-35571; Thermo Scientific), AlexaFluor 680-anti-mouse IgG (A-21057; Life Technologies).

Leupeptin and aprotinin were purchased from Roche. We used Na_3_VO_4_, PMSF, β-glycerophosphate, paraformaldehyde (PFA), cycloheximide (CHX), concanavalin A (ConA), BSA and NP40 (all from Sigma-Aldrich). Gö6976, PD98059 and MG-132 were from Calbiochem.

### Cell lines and mice

Human leukemic Jurkat T cells (American Type Culture Collection; ATCC) were maintained at subconfluence (<5 × 10^5^ cells/ml) in RPMI-1640 medium (BioWhittaker) supplemented with 10% FBS (Sigma or GBi Genycell Biotech) and 2 mM L-glutamine (Sigma or BioWhittaker) (37 °C, 5% CO2).

*Snx27* ^+/−^ mice were kindly provided by Dr. Wanjin Hong (Institute of Molecular and Cell Biology, Singapore)^24^. Mice were housed in specific pathogen-free conditions and handled in accordance with the Australian Code of Practice for the Care and Use of Animals for Scientific Purposes. All mouse strains and experimental protocols were conducted in accordance with the Animal Ethics Committee of the University of Queensland (approval #IMB/234/16/NHMRC/ARC and IMB/190/16/NHMRC/BREED).

### Isolation of primary T lymphocytes

Thymus, spleen, or peripheral lymph nodes were dissected and mechanically disaggregated in PBS. Single-cell suspensions were obtained using a 40-μm cell strainer (BD Biosciences). Splenocytes were treated with red blood cell (RBC) lysis buffer (eBioscience) according to manufacturer’s instructions. Cells were maintained in RPMI-1640 medium supplemented with 10% heat-inactivated FBS, 2 mM L-glutamine, penicillin/streptomycin (all from Gibco) and 50 mM β-mercaptoethanol (Sigma) (37 °C, 5% CO2).

### T cell activation assays

Stimulating conditions. Jurkat T cells were stimulated in complete medium (10^7^ cells/ml) with soluble anti-CD3 or -CD3/CD28 antibodies (1 μg/ml) for the indicated times. Where indicated, cells were pretreated for pharmacological inhibition with Gö6976 (100 nM) or PD98059 (50 μM) (37 °C, 30 min) prior to stimulation. Primary mouse lymphocytes were stimulated in complete medium (2.5 × 10^6^ cells/ml) with plate-bound anti-CD3 (plate coated with 2.5 μg/ml anti-CD3; 1 h, 37 °C); where indicated, medium was supplemented with anti-CD28 (1.25 μg/ml).

### Silencing experiments

For SNX27 and DGKζ silencing, pSUPER-derived plasmids were used as detailed in^19^.

### Dual luciferase reporter assays

Assays were performed as described in^19^. Briefly, Jurkat cells were transfected with the indicated shRNA constructs. At 24 h post-transfection, cells were washed and allowed to recover (24 h), then transfected with 15 μg of the indicated promoter construct (pGL2 AP-1 or pGL4-NF-κB) and 5 μg renilla luciferase vector pRL-TK (Promega) as internal control. After 24 h, cells were washed, allowed to recover (6 h) and stimulated as above. Cells were harvested and assayed for luciferase activity using the Dual-Luciferase Reporter Assay (Promega). Luciferase activity was reported relative to renilla luciferase activity (RLU).

### Proliferation assays

Cell proliferation was analyzed using the Cell Trace Violet Cell Proliferation kit (Invitrogen). Cells were stained according to manufacturer’s instructions, cultured for indicated times and processed for flow cytometry.

### Protein Stability Assays

Jurkat T cells were treated with 10μg/ml cycloheximide (CHX) alone, or in combination with MG-132 (5 μM) and abundance of the indicated proteins was assessed by quantitative western blot. Protein levels were normalized to GAPDH or α-tubulin.

### Western blot analysis

Cells were lysed in NP40 buffer (10 mM HEPES pH 7.5, 15 mM KCl, 1 mM EGTA, 1 mM EDTA, 1% NP40, 10% glycerol); clarified lysates were quantified with the Pierce 660 nm Protein Assay (Thermo Scientific). An equivalent protein amount per sample was analyzed by SDS-PAGE. Proteins were transferred to nitrocellulose membrane (Bio-Rad) and incubated with indicated primary antibodies. For HRP- or fluorescent-conjugated secondary antibodies, we used an ECL detection kit (Amersham Bioscience) or an Odyssey scanner (LI-COR), respectively. Densitometric analysis of proteins in western blots was performed using ImageJ.

### Flow cytometry analysis

Cells were collected in ice-cold PBS and cell surface proteins stained with saturating concentrations of the indicated fluorophore-conjugated primary antibodies in PBS staining buffer (1% FBS, 0.5% BSA, 0.01% sodium azide/PBS) (30 min, 4 °C). Cells were washed with the same buffer and fixed using 1% PFA/PBS or maintained at 4 °C for flow cytometry using Cytomics FC500 or Gallios cytometer (Beckman Coulter). Live cells were gated using forward and side scatter parameters. For fixed cells, LIVE/DEAD violet dead fixable cell stain (Invitrogen) was used. For primary cells, each sample was acquired for a minimum of 100,000 events. Data were analyzed using FlowJo software (TreeStar).

### IL-2 production assays

Jurkat T cells (2.5 × 10^5^ cells in 200 μl) were seeded in a flat bottom 96 well plate in triplicates, and stimulated as above, for 16 h at 37 °C. Then, ELISA test was performed on the culture supernatants, according to manufacturer’s instructions (Human IL-2 ELISA MAX^TM^ Delux, Biolegend). Primary T cells were stimulated as indicated under activation assays, after 48 hours supernatants were collected and IL-2 determined according to manufacturer’s instructions (Mouse IL-2 ELISA MAX^TM^ Delux, Biolegend)

### Statistical Analyses

Student’s t-test was used to analyze differences between two conditions, using the paired two-tailed t-test when comparing data sets from each pair of WT and *Snx27* ^−/−^ mouse littermates. Two-way ANOVA with the Bonferroni post-hoc test was used for multiple comparisons, both with GraphPad Prism 5 software. Differences were considered not significant (ns) when p > 0.05, significant (*) when p < 0.05, very significant (**) when p < 0.01 and extremely significant (***) when p < 0.001. or (****) when p < 0.0001.

## References

[1] Cullen PJ (2008). Endosomal sorting and signalling: an emerging role for sorting nexins. Nat Rev Mol Cell Biol.

[2] Teasdale RD, Collins BM (2012). Insights into the PX (phox-homology) domain and SNX (sorting nexin) protein families: structures, functions and roles in disease. Biochem J.

[3] Chan AS (2016). Sorting nexin 27 couples PTHR trafficking to retromer for signal regulation in osteoblasts during bone growth. Mol Biol Cell.

[4] Lauffer BE (2010). SNX27 mediates PDZ-directed sorting from endosomes to the plasma membrane. J Cell Biol.

[5] Steinberg F (2013). A global analysis of SNX27-retromer assembly and cargo specificity reveals a function in glucose and metal ion transport. Nat Cell Biol.

[6] Lunn ML (2007). A unique sorting nexin regulates trafficking of potassium channels via a PDZ domain interaction. Nat Neurosci.

[7] Clairfeuille T (2016). A molecular code for endosomal recycling of phosphorylated cargos by the SNX27-retromer complex. Nat Struct Mol Biol.

[8] Rincon E (2011). Translocation dynamics of sorting nexin 27 in activated T cells. J Cell Sci.

[9] Rincon E (2007). Proteomics identification of sorting nexin 27 as a diacylglycerol kinase zeta-associated protein: new diacylglycerol kinase roles in endocytic recycling. Mol Cell Proteomics.

[10] Merida I, Andrada E, Gharbi SI, Avila-Flores A (2015). Redundant and specialized roles for diacylglycerol kinases alpha and zeta in the control of T cell functions. Sci Signal.

[11] Andrada E (2016). Diacylglycerol kinase zeta limits the polarized recruitment of diacylglycerol-enriched organelles to the immune synapse in T cells. Sci Signal.

[12] Dower NA (2000). RasGRP is essential for mouse thymocyte differentiation and TCR signaling. Nat Immunol.

[13] Gharbi SI (2013). Transient PKCalpha shuttling to the immunological synapse is governed by DGKzeta and regulates L-selectin shedding. J Cell Sci.

[14] Kortum RL, Rouquette-Jazdanian AK, Samelson LE (2013). Ras and extracellular signal-regulated kinase signaling in thymocytes and T cells. Trends Immunol.

[15] Isakov N, Altman A (2012). PKC-theta-mediated signal delivery from the TCR/CD28 surface receptors. Frontiers in immunology.

[16] Sommer K (2005). Phosphorylation of the CARMA1 linker controls NF-kappaB activation. Immunity.

[17] Matsumoto R (2005). Phosphorylation of CARMA1 plays a critical role in T Cell receptor-mediated NF-kappaB activation. Immunity.

[18] Hamilton KS (2014). T cell receptor-dependent activation of mTOR signaling in T cells is mediated by Carma1 and MALT1, but not Bcl10. Sci Signal.

[19] Avila-Flores, A., Arranz-Nicolas, J., Andrada, E., Soutar, D. & Merida, I. Predominant contribution of DGKzeta over DGKalpha in the control of PKC/PDK-1-regulated functions in T cells. *Immunol Cell Biol*, 10.1038/icb.2017.7 (2017). 10.1038/icb.2017.7 28163304

[20] Gharbi SI (2011). Diacylglycerol kinase zeta controls diacylglycerol metabolism at the immunological synapse. Mol Biol Cell.

[21] Sancho D, Gomez M, Sanchez-Madrid F (2005). CD69 is an immunoregulatory molecule induced following activation. Trends Immunol.

[22] Zhong XP (2003). Enhanced T cell responses due to diacylglycerol kinase zeta deficiency. Nat Immunol.

[23] Riese MJ (2011). Decreased diacylglycerol metabolism enhances ERK activation and augments CD8+ T cell functional responses. J Biol Chem.

[24] Cai L, Loo LS, Atlashkin V, Hanson BJ, Hong W (2011). Deficiency of sorting nexin 27 (SNX27) leads to growth retardation and elevated levels of N-methyl-D-aspartate receptor 2C (NR2C). Mol Cell Biol.

[25] Love PE, Bhandoola A (2011). Signal integration and crosstalk during thymocyte migration and emigration. Nat Rev Immunol.

[26] Budd RC (1987). Distinction of virgin and memory T lymphocytes. Stable acquisition of the Pgp-1 glycoprotein concomitant with antigenic stimulation. J Immunol.

[27] Buck MD, O’Sullivan D, Pearce EL (2015). T cell metabolism drives immunity. J Exp Med.

[28] Waickman AT, Powell JD (2012). Mammalian target of rapamycin integrates diverse inputs to guide the outcome of antigen recognition in T cells. J Immunol.

[29] Kane LP, Andres PG, Howland KC, Abbas AK, Weiss A (2001). Akt provides the CD28 costimulatory signal for up-regulation of IL-2 and IFN-gamma but not TH2 cytokines. Nat Immunol.

[30] Cornish GH, Sinclair LV, Cantrell DA (2006). Differential regulation of T-cell growth by IL-2 and IL-15. Blood.

[31] Okkenhaug K (2002). Impaired B and T cell antigen receptor signaling in p110delta PI 3-kinase mutant mice. Science.

[32] Zheng Y (2007). A role for mammalian target of rapamycin in regulating T cell activation versus anergy. J Immunol.

[33] Pollizzi KN, Waickman AT, Patel CH, Sun IH, Powell JD (2015). Cellular size as a means of tracking mTOR activity and cell fate of CD4+ T cells upon antigen recognition. PloS one.

[34] Daley SR (2013). Rasgrp1 mutation increases naive T-cell CD44 expression and drives mTOR-dependent accumulation of Helios+ T cells and autoantibodies. eLife.

[35] Luo B, Prescott SM, Topham MK (2003). Protein kinase C alpha phosphorylates and negatively regulates diacylglycerol kinase zeta. J Biol Chem.

[36] Luo B, Prescott SM, Topham MK (2004). Diacylglycerol kinase zeta regulates phosphatidylinositol 4-phosphate 5-kinase Ialpha by a novel mechanism. Cell Signal.

[37] Jameson SC, Lee YJ, Hogquist KA (2015). Innate memory T cells. Adv Immunol.

[38] Abramovici H, Hogan AB, Obagi C, Topham MK, Gee SH (2003). Diacylglycerol kinase-zeta localization in skeletal muscle is regulated by phosphorylation and interaction with syntrophins. Mol Biol Cell.

[39] Gorentla BK, Wan CK, Zhong XP (2011). Negative regulation of mTOR activation by diacylglycerol kinases. Blood.

[40] Yang X, Xu T (2011). Molecular mechanism of size control in development and human diseases. Cell research.

[41] Gui J, Mustachio LM, Su DM (2012). & Craig, R. W. Thymus Size and Age-related Thymic Involution: Early Programming, Sexual Dimorphism, Progenitors and Stroma. Aging and disease.

[42] Chi H (2012). Regulation and function of mTOR signalling in T cell fate decisions. Nat Rev Immunol.

[43] Iglesias T (2000). Identification and cloning of Kidins220, a novel neuronal substrate of protein kinase D. J Biol Chem.

